# Supplementary material for: Structure Features and Physicochemical Performances of Fe-Contained Clinoptilolites Obtained via the Aqueous Exchange of the Balanced Cations and Isomorphs Substitution of the Heulandite Skeletons for Electrocatalytic Activity of Oxygen Evolution Reaction and Adsorptive Performance of CO2
Source: Molecules. 2023 Mar 23;28(7):2889. doi: 10.3390/molecules28072889 (PMC10095863; doi:10.3390/molecules28072889)
Supplement: Supplementary file 1 [file molecules-28-02889-s001.zip › molecules-2145851-supplementary.pdf]

Electronic Supporting Information

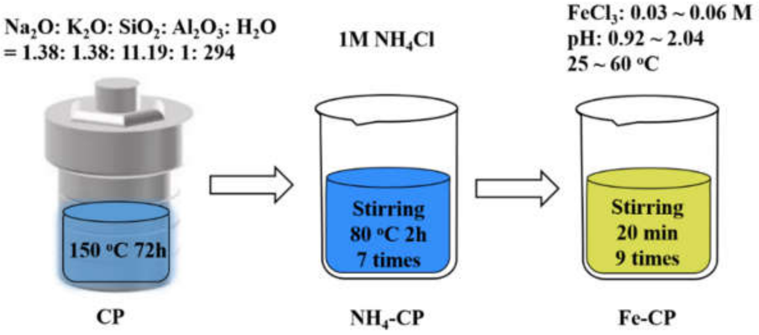

Scheme S1. Scheme 1. Schematic of the Fe-CP preparation.

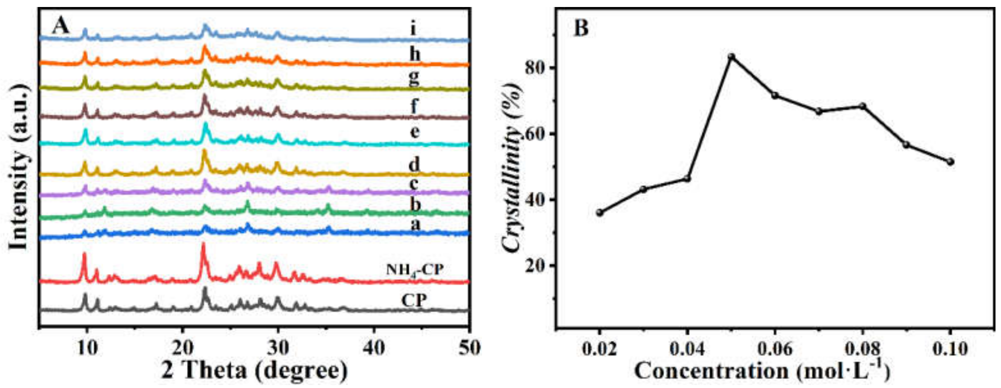

Figure S1. XRD patterns (A) and relative crystallinity of CP, NH4-CP, and Fe(0.03)-HCl(y)-CP (B). y = 0.02 (a), 0.03 (b), 0.04 (c), 0.05 (d), 0.06 (e), 0.07 (f), 0.08 (g), 0.09 (h), and 0.10 (i).

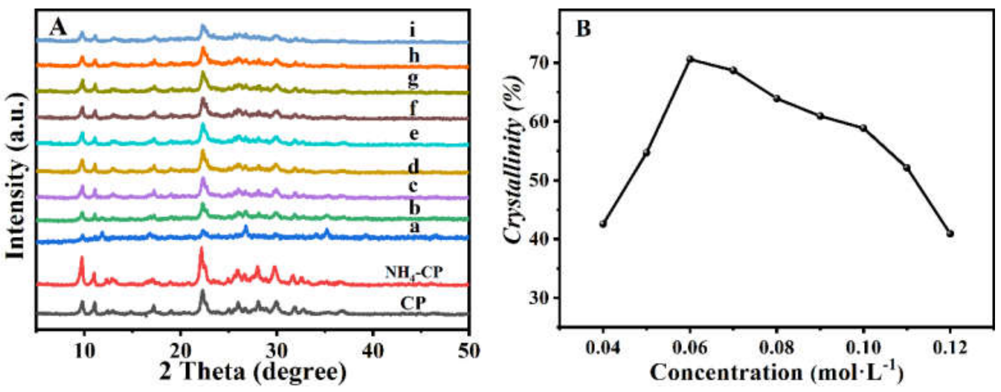

Figure S2. XRD patterns and relative crystallinity of CP, NH4-CP, and Fe(0.06)-HCl(y)-CP. y = 0.04 (a), 0.05 (b), 0.06 (c), 0.07 (d), 0.08 (e), 0.09 (f), 0.10 (g), 0.11 (h), and 0.12 (i), respectively.

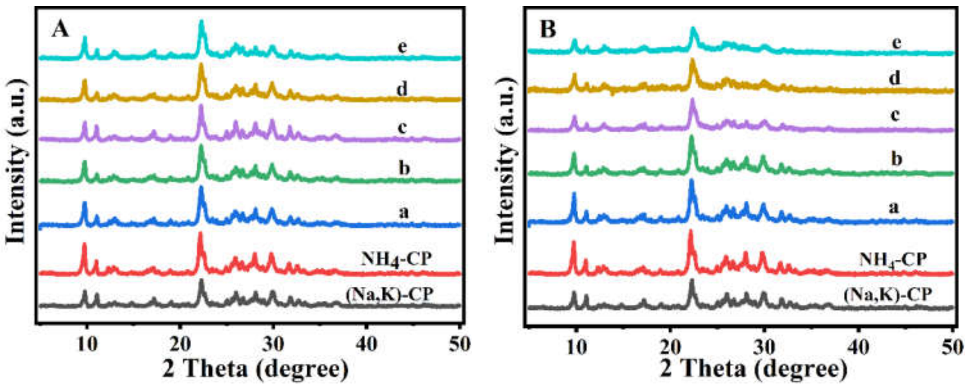

Figure S3. XRD patterns of CP, NH_4-CP, Fe(0.03)-HCl(0.05)-a-CP (A) and Fe(0.06)-HCl(0.07)-CP-a (B). a = 1 (a), 3 (b), 5 (c), 7 (d), and 9 (e), respectively.

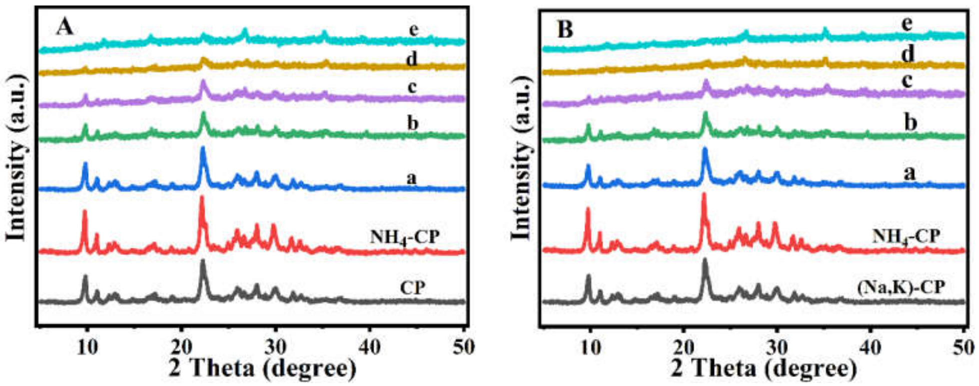

Figure S4. XRD patterns of CP, NH_4-CP, and Fe(0.03)-HCl(0.00)-CP-a (A) and Fe(0.06)-HCl(0.00)-CP-a (B). x = 1 (a), 3 (b), 5 (c), 7 (d), and 9 (e).

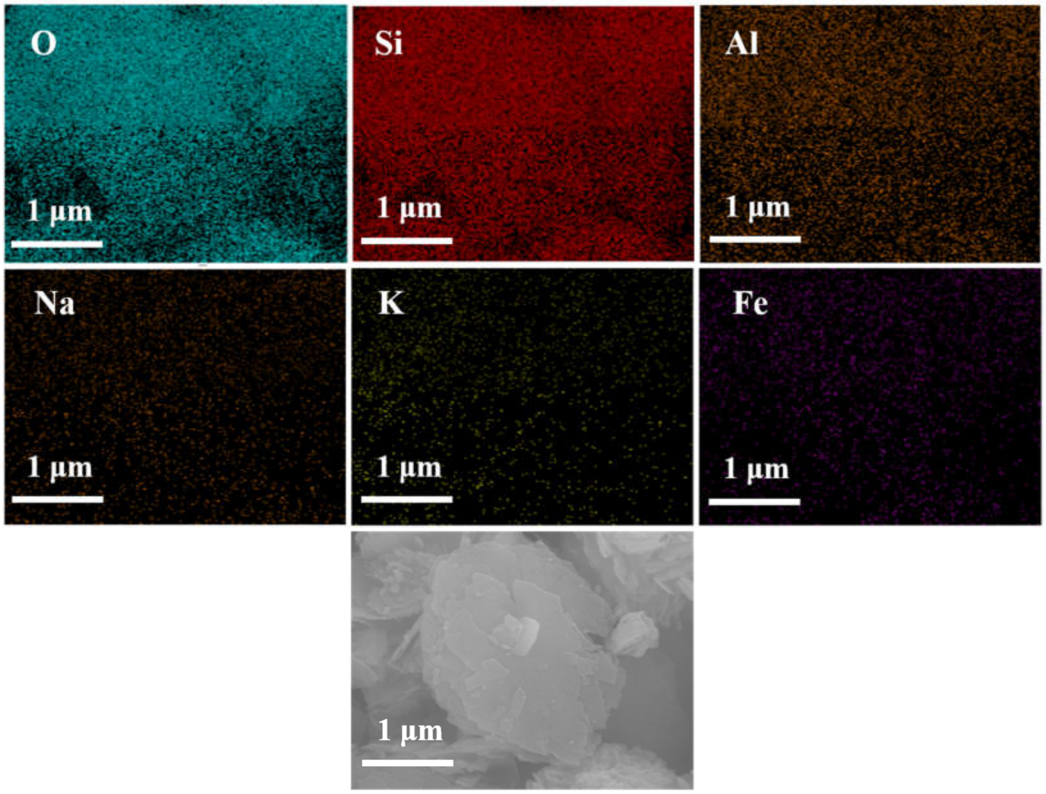

Figure S5. SEM elemental mapping of Fe(0.03)-HCl(0.00)-9-CP.

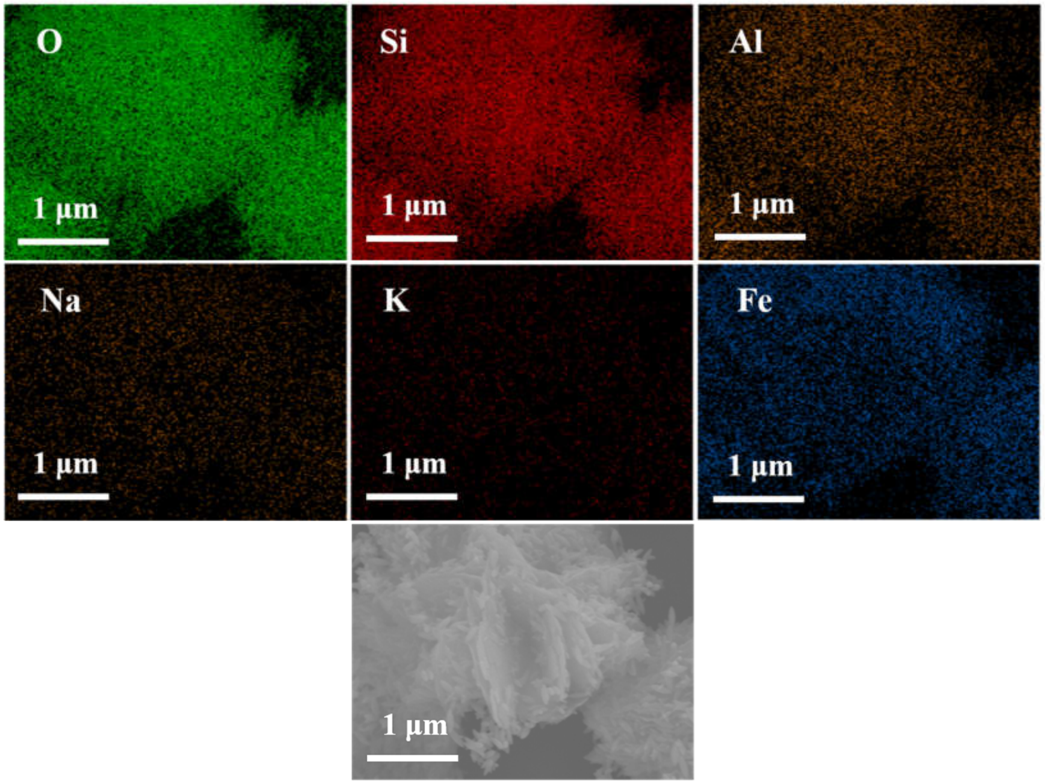

Figure S6. SEM elemental mapping of Fe(0.03)-HCl(0.05)-9-CP.

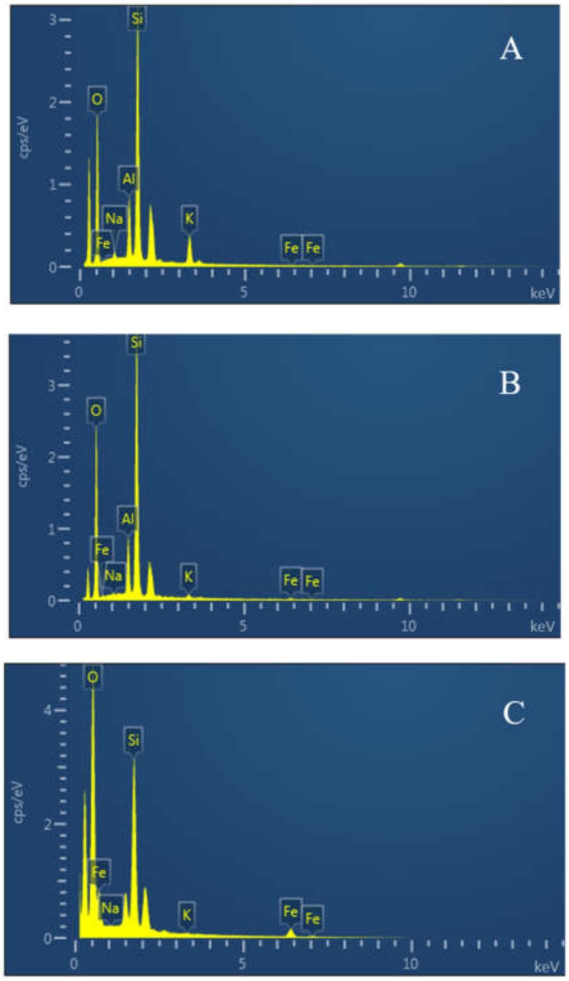

Figure S7. Energy dispersive X-ray (EDX) analysis of CP (A), Fe(0.03)-H(0.05)-CP-9 (B) and Fe(0.03)-H(0.00)-CP-9 (C).

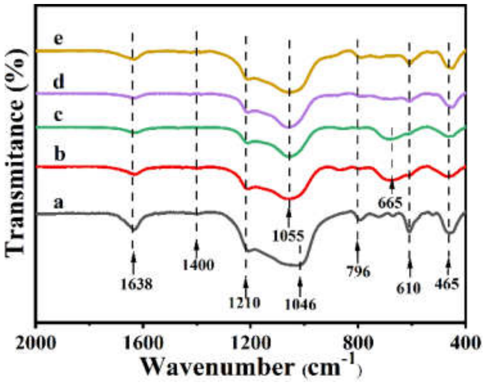

Figure S8. FT-IR spectra of various CPs. (a) CP, (b) Fe(0.03)-H(0.00)-CP-9, (c) Fe(0.06)-H(0.00)-CP-9, (d) Fe(0.03)-H(0.05)-CP-9, and (e) Fe(0.06)-H(0.07)-CP-9.

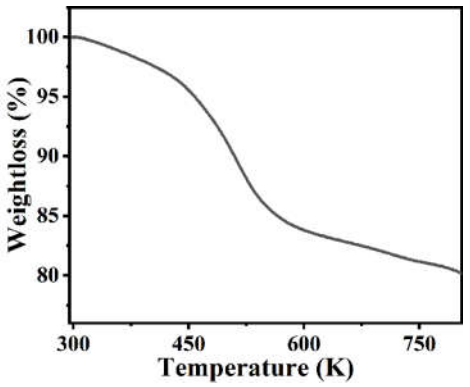

Figure S9. TG result for the bare FeOOH.

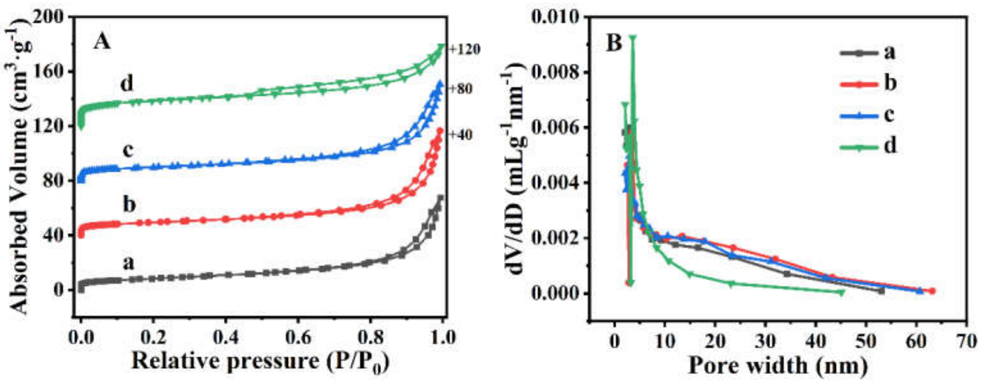

Figure S10. N_2 adsorption-desorption isotherms of (a) CP, (b) Fe(0.03)-H(0.05)-CP-3, (c) Fe(0.03)-H(0.05)-CP-9, and (d) Fe(0.03)-H(0.00)-CP-9, corresponding their mesopore size distribution (B).

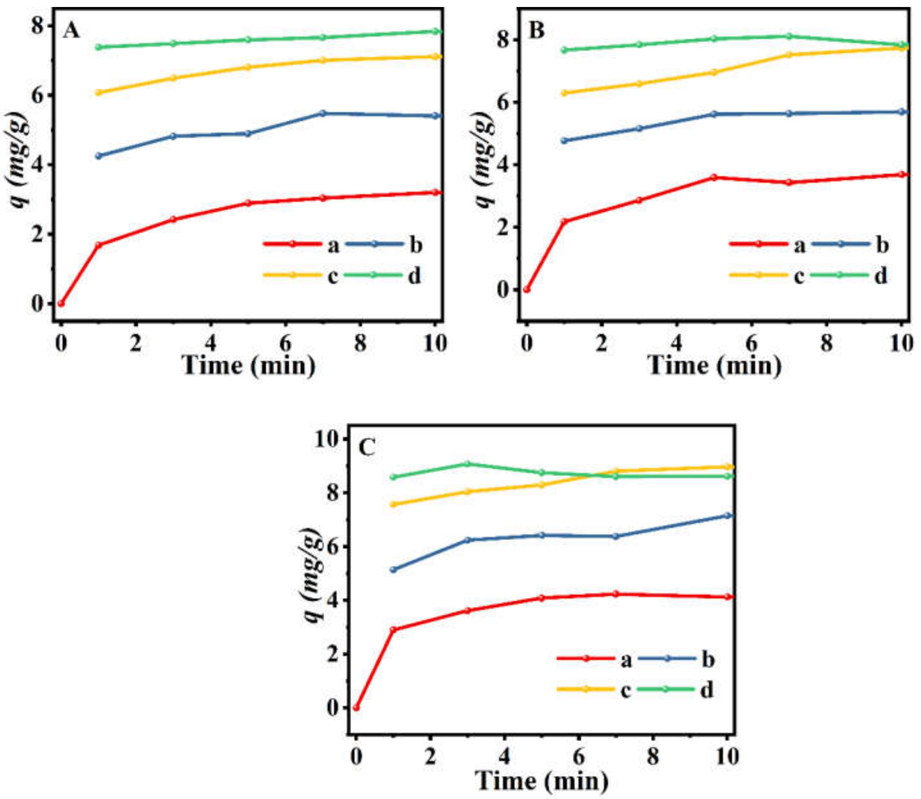

Figure S11. Kinetic curves of Fe(0.06)-H(0.07)-CP-x at 298 K (A), 313 K (B), and 333 K (C). x = 1 (a), 3 (b), 5 (c), 7 (d), and 9 (e).

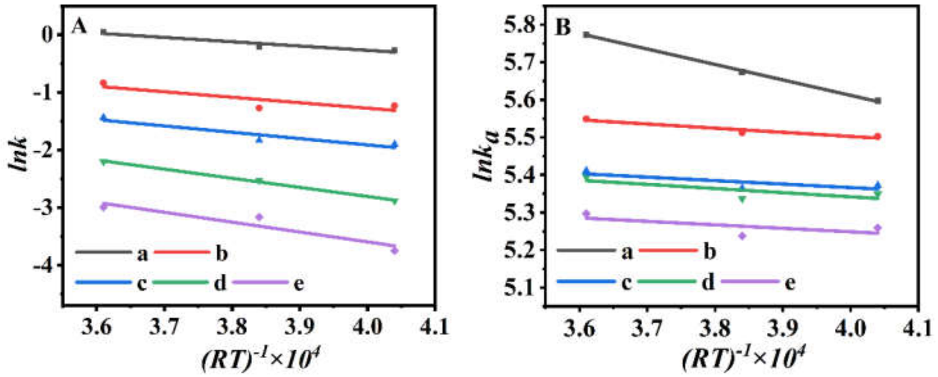

Figure S12. Relationships between $ln\ k$ and $(RT)^{-1} \times 10^4$ (A), as well as $ln\ K_a$ and $(RT)^{-1} \times 10^4$ (B) of Fe(0.03)-HCl(0.05)-x-CP. x = 1 (a), 3 (b), 5 (c), 7 (d), and 9 (e).

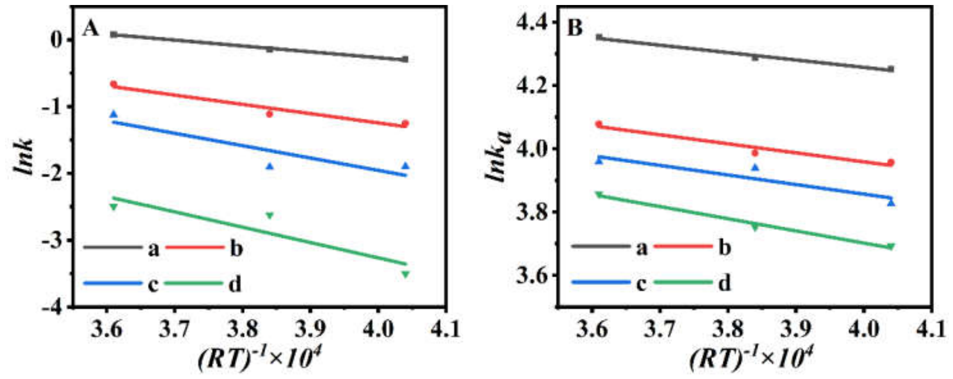

Figure S13. Relationships between $ln\ k$ and $(RT)^{-1} \times 10^4$ (A), as well as $ln\ k_a$ and $(RT)^{-1} \times 10^4$ (B) of Fe(0.06)-HCl(0.07)-x-CP. x = 1 (a), 3 (b), 5 (c), and 7 (d).

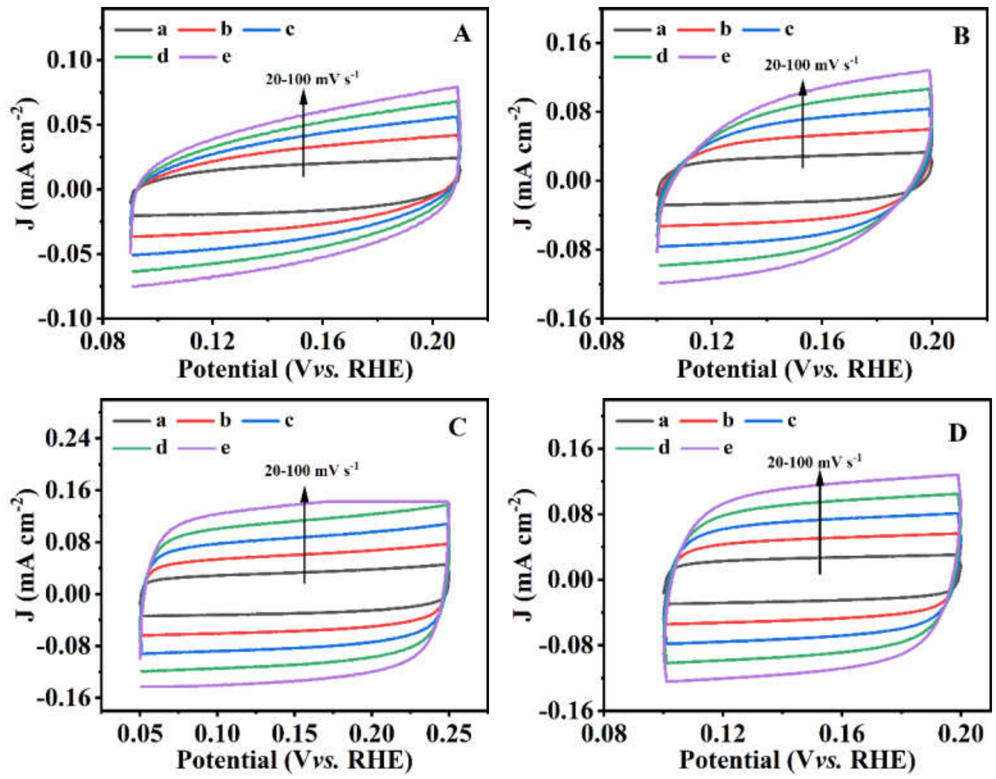

Figure S14. CV curves at different scan rates (20, 40, 60, 80, and 100 mV s^{-1} along the direction of the arrow) of (A) CP, (B) Fe(0.03)-HCl(0.05)-CP-3, (C) Fe(0.03)-HCl(0.05)-CP-9, and (D) Fe(0.03)-HCl(0.00)-CP-9.

Table S1. Summaries of various operation parameters of modification, the corresponding modified conditions, and the pH values of the system.

sample code	Concentration (mol/L)		Temperature (K)	pH value
	$FeCl_3$	HCl		
Fe(0.03)-H(0.00)-CP	0.03	0.00	333	2.04
Fe(0.03)-H(0.02)-CP	0.03	0.02	333	1.66
Fe(0.03)-H(0.03)-CP	0.03	0.03	333	1.50
Fe(0.03)-H(0.04)-CP	0.03	0.04	333	1.38
Fe(0.03)-H(0.05)-CP	0.03	0.05	333	1.31
Fe(0.03)-H(0.06)-CP	0.03	0.06	333	1.25
Fe(0.03)-H(0.07)-CP	0.03	0.07	333	1.18
Fe(0.03)-H(0.08)-CP	0.03	0.08	333	1.12
Fe(0.03)-H(0.09)-CP	0.03	0.09	333	1.04
Fe(0.03)-H(0.10)-CP	0.03	0.10	333	0.98
Fe(0.06)-H(0.00)-CP	0.06	0.00	333	1.85
Fe(0.06)-H(0.04)-CP	0.06	0.04	333	1.32
Fe(0.06)-H(0.05)-CP	0.06	0.05	333	1.26
Fe(0.06)-H(0.06)-CP	0.06	0.06	333	1.18
Fe(0.06)-H(0.07)-CP	0.06	0.07	333	1.13
Fe(0.06)-H(0.08)-CP	0.06	0.08	333	1.09
Fe(0.06)-H(0.09)-CP	0.06	0.09	333	1.03
Fe(0.06)-H(0.10)-CP	0.06	0.10	333	1.01
Fe(0.06)-H(0.11)-CP	0.06	0.11	333	0.96
Fe(0.06)-H(0.12)-CP	0.06	0.12	333	0.92

Table S2. Compositions of CP, Fe(0.03)-H(0.05)-CP-9 and Fe(0.03)-H(0.00)-CP-9 obtained via EDX quantitative analysis.

	Fe at.%	O at.%	Si at.%	Al at.%	Na at.%	K at.%
CP	0.00	63.90	28.51	6.52	1.07	5.05
Fe(0.03)-H(0.05)-CP-9	0.55	62.95	30.26	6.19	0.00	0.00
Fe(0.03)-H(0.00)-CP-9	9.57	60.97	23.10	6.34	0.00	0.00

Table S3. Summary of textural properties for various samples

Sample	BET surface area ($m^2·g^{-1}$)	Micropore surface area ($m^2·g^{-1}$)	External surface area ($m^2·g^{-1}$)	Micropore Volume ($mL·g^{-1}$)
CP	28.749	4.274	24.476	0.003
Fe(0.03)-H(0.05)-CP-3	33.364	10.111	23.253	0.005
Fe(0.03)-H(0.05)-CP-9	34.921	12.081	22.840	0.006
Fe(0.03)-H(0.00)-CP-9	67.003	40.670	26.333	0.018

Table S4. Summaries for $\Delta_r G^{\theta}_m$ (kJ/mol), $\Delta_r S^{\theta}_m$ (J/mol k), $\Delta_r H^{\theta}_m$ (kJ/mol), and E_a (kJ) values of various modified process with NH_4-CP.

Samples	Exchange Times	298K		313K		333K		$\Delta_r H^{\theta}_m$	E_a
		$\Delta_r G^{\theta}_m$	$\Delta_r S^{\theta}_m$	$\Delta_r G^{\theta}_m$	$\Delta_r S^{\theta}_m$	$\Delta_r G^{\theta}_m$	$\Delta_r S^{\theta}_m$		
Fe(0.06)-H(0.07)-CP-x	1	-4.67	2.47	-4.92	2.47	-5.32	2.47	2.75	7.45
	3	-4.42	1.89	-4.65	1.97	-4.89	2.05	1.27	9.28
	5	-4.39	1.93	-4.60	2.00	-4.96	2.12	1.41	10.96
	7	-4.42	1.92	-4.63	1.99	-4.91	2.09	1.35	15.89
	9	-4.34	1.92	-4.52	1.98	-4.79	2.07	1.42	17.12
Fe(0.06)-H(0.07)-CP-x	1	-3.51	2.36	-3.72	2.43	-4.02	2.53	3.58	8.78
	3	-3.27	2.12	-3.46	2.18	-3.76	2.29	3.09	13.87
	5	-3.16	2.06	-3.42	2.15	-3.65	2.23	3.02	18.49
	7	-3.05	1.47	-3.25	1.54	-3.56	1.64	1.35	22.95

Table S5. Summaries of overpotentials (η) at 10 mA cm^{-2} and tafel slopes in 1.0 M KOH solution for OER properties obtained in this work and reported literature.

catalysts	η (mV)	Tafel slope (mV dec^{-1})	Reference
Fe(0.03)-H(0.05)-CP-9	560	129	this work
Fe(0.03)-H(0.00)-CP-9	510	79	this work
commercial RuO_2	330	76.3	[1]
$Co_{0.89}Fe_{0.11}O$-N	304	52.7	[1]
MIL-53(Fe)	233	88.7	[2]
CoNiFe ZIF-NFs	273	87	[3]
Fe-Co-CN/rGO	308	138	[4]
FeO_xCF-8	408	93	[5]
$CoFe_2O_4$/biocarbon	417	--	[6]

References

[1] Du, Q.; Su, P.; Cao, Z.; Yang, J.; Price, C.; Liu, J. Construction of N and Fe co-doped CoO/Co_xN interface for excellent OER performance. *Catal. Sci. Technol.* **2021**, 10.1016/j.susmat.2021.e00293.

[2] Nivetha, R.; Kollu, P.; Chandar, K.; Pitchaimuthu, S.; Jeong, S.; Grace, A. Role of MIL-53(Fe)/hydrated–dehydrated MOF catalyst for electrochemical hydrogen evolution reaction (HER) in alkaline medium and photocatalysis. *RSC advances* **2019**, *9*, 3215–3223.

[3] Sankar S.; Manjula K.; Keerthana G.; Babu B.; Kundu S. Highly stable trimetallic (Co, Ni, and Fe) zeolite imidazolate framework microfibers: an excellent electrocatalyst for water oxidation. *Cryst. Growth Des.* **2021**, *21*, 1800–1809.

[4] Fang W.; Wang J.; Hu Y.; Cui Q.; Zhu R.; Zhang Y.; Yue C.; Dang J.; Cui W.; Zhao H.; Li Z. Metal-organic framework derived Fe-Co-CN/reduced graphene oxide for efficient HER and OER. *Electrochim. Acta.* **2021**, *365*, 10.1021/acsami.7b08647.

[5] Yan F.; Zhu C.; Wang S.; Zhao Y.; Zhang X.; Chen Y. Electrochemically activated-iron oxide nanosheet arrays on carbon fiber cloth as a three-dimensional self-supported electrode for efficient water oxidation. *J. Mater. Chem. A.* **2016**, *4*, 6048–6055.

[6] Liu S.; Bian W.; Yang Z.; Tian J.; Jin C.; Shen M.; Zhou Z.; Yang R. A facile synthesis of CoFe2O4/biocarbon nanocomposites as efficient bi-functional electrocatalysts for the oxygen reduction and oxygen evolution reaction. *J. Mater. Chem. A.* **2014**, *2*, 18012–18017.
